# Dataset for the proteomic and transcriptomic analyses of perivitelline fluid proteins in *Pomacea* snail eggs

**DOI:** 10.1016/j.dib.2017.09.020

**Published:** 2017-09-22

**Authors:** Huawei Mu, Jin Sun, Horacio Heras, Ka Hou Chu, Jian-Wen Qiu

**Affiliations:** a Department of Biology, Hong Kong Baptist University, Hong Kong, China; b Division of Life Science, Hong Kong University of Science and Technology, Hong Kong, China; c Instituto de Investigaciones Bioquímicas de La Plata (INIBIOLP), Universidad Nacional de La Plata (UNLP)-CONICET CCT-La Plata, La Plata, Argentina; d Cátedra de Química Biológica, Facultad de Ciencias Naturales y Museo, UNLP, Argentina; e Simon F.S. Li Marine Science Laboratory, School of Life Sciences, The Chinese University of Hong Kong, Hong Kong, China

**Keywords:** Apple snail, Perivitelline fluid, Proteome, Transcriptome

## Abstract

This article describes how the proteomic and transcriptomic data were produced during a study of the reproductive proteins of *Pomacea maculata*, an aquatic apple snail laying colorful aerial eggs, and provides public access to the data. The data are related to a research article titled ‘An integrated proteomic and transcriptomic analysis of perivitelline fluid proteins in a freshwater gastropod laying aerial eggs’ (Mu et al., 2017) [1]. RNA was extracted from the albumen gland and other tissues and sequenced on an Illumina Hiseq. 2000. The assembled transcriptome was translated into protein sequences and then used for protein identification. Proteins from the perivitelline fluid of *P. maculata* were separated in SDS-PAGE and analyzed by LTQ-Orbitrap Elite coupled to an Easy-nLC. The translated transcriptome data are provided in this article. Proteomic data (.*raw* file format) are available via ProteomeXchange with the identifier PXD006718.

**Specifications Table**

**Table:** 

Subject area	Biology
More specific subject area	Apple snail proteomics and transcriptomics
Type of data	Table and.*raw* file
How data was acquired	SDS-PAGE, strong cation exchange (SCX) chromatography, and LTQ-Orbitrap Elite coupled to an Easy-nLC were used to acquire the proteomic data;
	Illumina Hiseq. 2000 sequencing was applied to acquire the transcriptomic data.
Data format	Analyzed
Experimental factors	Perivitelline fluid of snail eggs laid within 12 h.
Experimental features	Mass spectrometry was applied to determine the proteome profile of the egg perivitelline fluid, and transcriptome sequencing was used to determine differential gene expression in different tissues.
Data source location	Department of Biology, Hong Kong Baptist University, Hong Kong, China
Data accessibility	Data are available via ProteomeXchange with the identifier PXD006718
Related research article	An integrated proteomic and transcriptomic analysis of perivitelline fluid proteins in a freshwater gastropod laying aerial eggs [1]

**Value of the data**

• This dataset provides a comprehensive proteomic profile of perivitelline fluid of the apple snail *Pomacea maculata*. The proteomic data which were obtained from state-of-the-art mass spectrometry analysis can be used for protein identification, especially for reproductive proteins in gastropods.
• This dataset also provides translated transcriptomic profiles of the albumen gland and other tissues of *Pomacea maculata*. The translated transcriptome can be used as the database to support protein identification in gastropods.
• The data presented here can be used for studies of protein function and evolution in gastropods.

## 1 Data

*Pomacea maculata* is a freshwater snail native to South America that has invaded many regions of the world [2]. There is considerable interest in the reproductive biology of this species [3], [4], but a lack of genomic resources has hindered such studies at the molecular level. We extracted the RNA from the albumen gland and other tissues, and sequenced them on Illumina Hiseq. 2000 to generate a database to support protein identification. Table 1 shows the number of contigs and unigenes in the assembled transcriptome, as well as the quality of the data. Table S2 contains 44,350 protein sequences which were translated from the transcriptome. These sequences were used for protein identification as described below. Proteins were extracted from the perivitelline fluid of newly laid eggs, fractionated using SDS-PAGE and analyzed with LTQ-Orbitrap Elite coupled to an Easy-nLC. The data files (.*raw*) generated by mass spectrometry was converted into.*mgf* files using Proteome Discovery 1.3.0.339 and searched against the protein database in Mascot 2.3.2 and they were deposited in ProteomeXchange.

**Table 1:** Statistics of transcriptome assembly quality of albumen gland (AG) and other tissues (OT) in *Pomacea maculata*.

	Sample name	Total Number	Total Length(nt)	Mean Length(nt)	N50	Total Consensus Sequences^a^	Distinct Clusters^b^	Distinct Singletons^c^
Contig	Pm_AG	179,342	52,426,027	292	427	–	–	–
	Pm_OT	211,148	72,423,210	343	557	–	–	–
								
Unigene	Pm_AG	92,567	52,283,278	565	878	92,567	13,896	78,671
	Pm_OT	130,305	81,931,694	629	1109	130,305	27,438	102,867
	Pm_AG&OT	105,349	82,687,751	785	1332	105,349	26,056	79,293

a All the assembled unigenes.

b The cluster unigenes; The same cluster contains many high similar unigenes which have more than 70% of similarity, and these unigenes may come from homologous or same gene.

c Distinct Singletons represents this unigene come from a single gene.

## 2 Experimental design, materials and methods

### 2.1 Animal culture

The *Pomacea maculata* adults originally collected from a river in San Pedro, Argentina (33°39′35.97″ S, 59°41′52.86″ W) were transported to Hong Kong Baptist University and cultured at 25±1 °C with dechlorinated tap water. Fish food, lettuce and carrot were fed to the snails. Egg clutches deposited by the snails on the walls of aquaria were used for protein extraction.

### 2.2 RNA extraction and transcriptome sequencing

In order to establish a database for protein identification and detect tissue specific genes, transcriptomes of albumen gland (AG) and other tissue (OT; including foot, mantle and visceral mass) were sequenced. Total RNA of AG and OT was extracted using TRIzol reagent (Invitrogen, Carlsbad, USA) following the manufacturer's protocol except two minor modifications: A mixed solution of 0.8 M Na_3_C_6_H_5_O_7_ and 1.2 M NaCl was added before the isopropanol step; A LiCl solution (final concentration 2 M) was added after resuspension of RNA pellets with RNase-free water. The messenger RNA was collected and reverse-transcribed into cDNA, and sequenced on an Illumina Hiseq. 2000 to produce 100 base pair of pair-ended reads. Clean reads were assembled using Trinity (release 20130225) [5]. The assembly statistics of the AG and OT transcriptomes are showed in Table 1. Assembled sequences were annotated using BLASTx by searching against public databases (NCBI nr, Swissprot, COG and KEGG) with an *E*-value threshold of 1×*e*^−^^5^ [6], [7]. Amino acid sequences were translated from the assembled sequences and used as the database for protein identification (Table 2).

### 2.3 Egg mass collection, protein extraction and mass spectrometry

Egg masses were washed with MilliQ water and then air-dried. A sterile needle was used to crack the egg shells gently and a pipette with a fine tip was used to collect the perivitelline fluid (PVF). PVF was stored in 8 M urea, homogenized, and centrifuged. Supernatant solution was collected, purified, and protein concentration was determined using RC-DC kit (Bio-Rad). There were three biological replicates which were collected from different egg masses.

The protein solutions were mixed with a SDS-PAGE buffer (0.05% bromophenol blue, 50% glycerol, 10 mM dithiothreitol, 0.2 M Tris–HCl pH=6.8, and 10% SDS) with a ratio of 3:1 (v/v), heated at 105 °C for 5 min, and separated by SDS-PAGE. Sample gels were stained with Coomassie Brilliant Blue and destained with 1% acetic acid and MilliQ. Each biological replicate was divided into 10 fractions. For each fraction, gels were cut into small pieces and further destained with a mixed solution of 50% methanol and 50 mM NH_4_HCO_3_, and then washed with MilliQ, 100% ACN, and 100 mM NH_4_HCO_3_ sequentially. Then 10 mM of dithiothreitol was applied to reduce the disulfide bonds, and 55 mM of iodoacetamide was used to alkylate the sulfhydryl groups. Each gel fraction was then digested using sequencing grade trypsin in 50 mM NH_4_HCO_3_ for 16 h. The peptide solutions were recovered, desalted with Sep-Pak C18 cartridges, and dried in a vacuum concentrator.

Each fraction from the three biological samples was reconstituted using 0.1% formic acid and analyzed twice with a LTQ-Orbitrap Elite coupled to an Easy-nLC as described previously [8]. In short, peptides from each fraction were separated in a C18 capillary column. Mass spectrometry scans over a range of 350–1600 *m*/*z* were conducted with a resolution of 60,000 under the positive charge mode. The top five abundant multiple-charged ions which had a minimum signal threshold of 500.0 were selected for fragmentation using collision-induced dissociation (CID) and high-energy collision-induced dissociation (HCD). Both CID and HCD scanning strategies used an isolation width of 2.0 *m*/*z*. The CID fragmentation adopted an activation time of 10 ms and a normalized collision energy of 35%; The HCD fragmentation also used an activation time of 10 ms but the normalized collision energy was 45%.

### 2.4 Protein identification

The raw MS/MS files were converted into.*mgf* files (Raw data are available via ProteomeXchange with identifier PXD006718) using Proteome Discovery 1.3.0.339, and searched against the *P. maculata* database with 77584 protein sequences containing both ‘decoy’ and ‘target’ sequences using Mascot version 2.3.2. The parameters were similar to those described in Mu et al. [9] except that the fixed modification was set as cysteine carbamidomethylation and the maximum number of missed cleavage of trypsin was set as one. Peptides having an ion score ≥22 (corresponding to 95% confidence) were kept. Peptides which had more than nine amino acids were retained and 1% of false discovery rate threshold was adopted in the protein identification. Proteins which had at least three matched peptides and were detected in at least two replicates were kept.
