# Supplementary material for: Dataset for the proteomic and transcriptomic analyses of perivitelline fluid proteins in Pomacea snail eggs
Source: Data Brief. 2017 Sep 22;15:203–7. doi: 10.1016/j.dib.2017.09.020 (PMC5633349; doi:10.1016/j.dib.2017.09.020)
Supplement: Supplementary file 1 — Transparency document [file mmc1.docx]

**Author declaration**

All the authors declare that there is no conflict of interest.
